# Estimation of Urea Alone in Urine

**Published:** 1914-05

**Authors:** 


ESTIMATION OF UREA ALONE IN URINE. Lematter, *Dresse Med.,* August 16, 1913. (Note: Pure uric acid and other nitrogenous constituents of the urine but not, we believe, proteids and nucleo-proteids, yield nitrogen on treatment with sodium hypobromite. Hence, so-called urea determinations in the urine are really approximate estimates of total non-proteid nitrogen. It would, perhaps, be better for clinical estimations to have the urea tubes graduated so as to show the amount of N by weight instead of the urea, 28/60 of which is nitrogen.—Ed.) 30 c.c. of urine and 50 c.c. of a fresh 30% phospho-tungstic acid solution are introduced into a 100 c.c. flask. Agitate and let stand 10 minutes; add 4 grammes of Magnesium chlorid. Add water to the 100 c.c. mark. Let stand till the precipitate has completely settled. Filter only the decantate as the precipitate will pass through a filter. Neutralize 10 c.c. (corresponding to 3 c.c of urine) with normal sodium hydroxid, to a permanent pink tinge with phenolphthalein. Then estimate the urea in the usual way.

